# Theoretical Verification of Photoelectrochemical Water Oxidation Using Nanocrystalline TiO_2_ Electrodes

**DOI:** 10.3390/molecules20069732

**Published:** 2015-05-27

**Authors:** Shozo Yanagida, Susumu Yanagisawa, Koichi Yamashita, Ryota Jono, Hiroshi Segawa

**Affiliations:** 1 Frontier Research Institute, Osaka University, 2-1, Yamada-oka, Suita, Osaka 565-0871, Japan; 2 Department of Physics and Earth Sciences, Faculty of Science, University of the Ryukyus, 1, Senbaru, Nishihara, Okinawa 903-0213, Japan; E-Mail: shou@sci.u-ryukyu.ac.jp; 3 Department of Chemical System Engineering, School of Engineering, The University of Tokyo, 7-3-1 Hongo, Bunkyo-ku, Tokyo 113-8656, Japan; E-Mails: yamasita@chemsys.t.u-tokyo.ac.jp (K.Y.); jono@tcl.t.u-tokyo.ac.jp (R.J.); 4 Research Center for Advance Science and Technology, The university of Tokyo, 4-6-1, Komaba, Meguro-ku, Tokyo 153-8904, Japan; E-Mail: csegawa@mail.ecc.u-tokyo.ac.jp

**Keywords:** DFT, HOMO, LUMO, spin density, conductivity, TiO_2_ photocatalysis, DSC, Honda/Fujishima effect

## Abstract

Mesoscopic anatase nanocrystalline TiO_2_ (nc-TiO_2_) electrodes play effective and efficient catalytic roles in photoelectrochemical (PEC) H_2_O oxidation under short circuit energy gap excitation conditions. Interfacial molecular orbital structures of (H_2_O)_3_ &OH(TiO_2_)_9_H as a stationary model under neutral conditions and the radical-cation model of [(H_2_O)_3_&OH(TiO_2_)_9_H]^+^ as a working nc-TiO_2_ model are simulated employing a cluster model OH(TiO_2_)_9_H (Yamashita/Jono’s model) and a H_2_O cluster model of (H_2_O)_3_ to examine excellent H_2_O oxidation on nc-TiO_2_ electrodes in PEC cells. The stationary model, (H_2_O)_3_&OH(TiO_2_)_9_H reveals that the model surface provides catalytic H_2_O binding sites through hydrogen bonding, van der Waals and Coulombic interactions. The working model, [(H_2_O)_3_&OH(TiO_2_)_9_H]^+^ discloses to have a very narrow energy gap (0.3 eV) between HOMO and LUMO potentials, proving that PEC nc-TiO_2_ electrodes become conductive at photo-irradiated working conditions. DFT-simulation of stepwise oxidation of a hydroxide ion cluster model of OH^−^(H_2_O)_3_, proves that successive two-electron oxidation leads to hydroxyl radical clusters, which should give hydrogen peroxide as a precursor of oxygen molecules. Under working bias conditions of PEC cells, nc-TiO_2_ electrodes are now verified to become conductive by energy gap photo-excitation and the electrode surface provides powerful oxidizing sites for successive H_2_O oxidation to oxygen via hydrogen peroxide.

## 1. Introduction

Photoelectrochemical (PEC) water (H_2_O) oxidation on TiO_2_ electrodes was qualitatively explained as due to downward band bending induced by depletion layer of TiO_2_ rutile crystal electrodes by assuming the energy structure of TiO_2_, e.g., conduction band potential (E_cb_) of −0.65 V(SCE) and valence band potential (E_vb_) of 2.35 V (SCE), energy gap 3.0 eV, and 1.23 eV of the equilibrium cell potential for H_2_O electrolysis at 25 °C and 1 atmospheric pressure [1,2]. We noticed recently that efficient photoelectrochemical H_2_O oxidation using anatase nc-TiO_2_ electrodes was at first reported in 1987 by Sakka *et al*. [3]. Interestingly, they reported vigorous oxygen (O_2_) and hydrogen (H_2_) evolution using acidic aqueous solution (0.1 N H_2_SO_4_) at the PEC nc-TiO_2_ cell. It is worth noting that the notable electron flow due to H_2_O oxidation to O_2_ becomes detectable when bias potential reaches at about 2.0 V (*vs*. SCE), and that under energy gap UV irradiation, photocurrent starts to flow at bias potential around −0.5~−0.3 V (*vs*. SCE), showing vigorous O_2_ and H_2_ evolution at bias potential around 0.5–1.0 V (*vs*. SCE). Such effective acidic H_2_O oxidation on mesoporous nc-TiO_2_ electrodes prompts us to understand the H_2_O photooxidation on the basis of molecular orbital (MO) theory, because the band-bending concept is based on crystal-level physics, and nc-TiO_2_ in PEC electrodes is too small to form depletion layer in nc-TiO_2_ with average size of 25 nm.

Computational chemistry using density functional theory (DFT) well explains and predicts molecular energy structures and properties functioned by self-association of molecules where hydrogen bonding or van der Waals and Coulombic interactions as non-covalent bonding play an essential role [4,5,6,7,8,9]. DFT calculations using the nc-TiO_2_ model of Ti_9_O_18_H-OH (Yamashita/Jono model) successfully verified that the surface complex between nc-TiO_2_ and 7,7,8,8-tetracyanoquinodimenthane shows charge transfer transition [10]. We now report verification of PEC oxidation of H_2_O on nc-TiO_2_ electrodes on the basis of DFT simulation using Yamashita/Jono nc-TiO_2_ model. Here, DFT-simulations verify that H_2_O forms H_2_O clusters via hydrogen bonding and that the H_2_O clusters-associated nc-TiO_2_ electrodes provide excellent H_2_O oxidation sites. In addition, the working models of H_2_O clusters-associated PEC-nc-TiO_2_ electrodes are simulated as radical cations, and effective PEC H_2_O oxidation is verified as well theoretically.

## 2. Results and Discussion

### 2.1. Yamashita/Jono Model for Simulation of PEC-nc-TiO_2_ Electrodes

**Figure 1:**
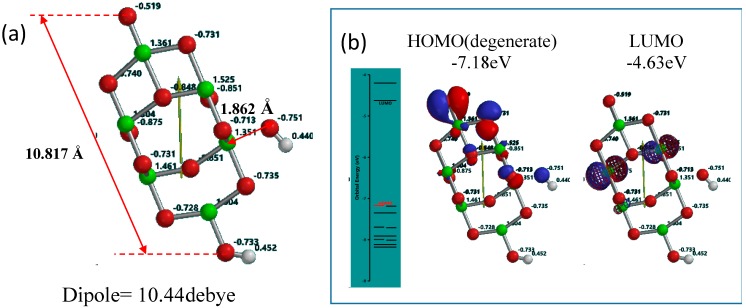
Yamashita/Jono model of OH(TiO_2_)_9_H as a model of PEC-nc-TiO_2_ electrodes, (**a**) Size, distance and Mulliken charge; (**b**) the energy structure and configuration of HOMO and LUMO.

Yamashita and Jono’s anatase nc-TiO_2_ model (Ti_9_O_18_H-OH) consists of nine TiO_2_ units (TiO_2_)_9_ derived from the packing unit of the crystalline anatase TiO_2_, hydroxide on surface side of the TiO_2_ and hydrogen on one side of the TiO_2_. To optimize the non-covalent distance between hydroxyl group and nc-TiO_2_ unit, all heavy atoms of the nc-TiO_2_ unit are frozen and the model was simulated to the energetically optimized geometry (see Supplementary Figure S1). The distance (1.862 Å) become shorter, and the refined Yamashita/Jono model is abbreviated as OH(TiO_2_)_9_H hereafter and size of about 1 nm length, Mulliken charge, the energy structures and configurations of the highest occupied molecular orbital (HOMO) and the lowest unoccupied molecular orbital (LUMO) are shown in Figure 1.

Mulliken charge in OH(TiO_2_)_9_H indicates that hydroxyl group is charged negative and the protonated nc-TiO_2_ unit positive, and then we regard that Yamashita/Jono model is a kind of ion-dipole complex or weak charge transfer complex. The HOMO distributes exclusively on hydroxide ion and the LUMO inside of the nc-TiO_2_ unit, giving energy gap 2.55 eV. To know self-association of Yamashita/Jono model, the OH and H-detached (TiO_2_)_9_ and (TiO_2_)_9_H units are both DFT-simulated as charge is neutral and cation, respectively (Supplementary Tables S1 and S2 and Figures S1 and S2).

**Figure 2:**
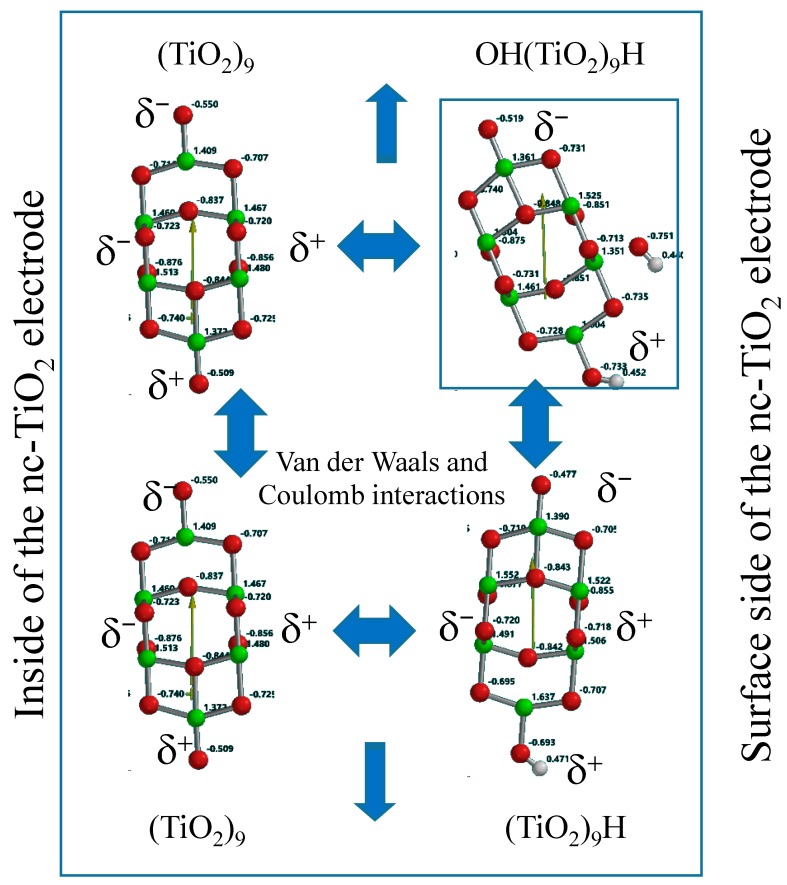
Association of Yamashita/Jono model via van der Waals and Coulomb interactions, explaining growth of the model to larger size PEC-nc-TiO_2_ electrodes.

The DFT simulation data reveals that Mulliken charge on the hydrogen-bearing side is positive and another side negative as well as the OH(TiO_2_)_9_H model. Further, the HOMO and LUMO are almost degenerate. The association through van der Waals and Coulombic interaction and the comparable dipole to that of the Yamashita/Jono model support that Yamashita/Jono model may self-associate with (TiO_2_)_9_ and (TiO_2_)_9_H to yield surface thin-film anatase TiO_2_ electrodes as depicted in Figure 2.

### 2.2. Water Cluster Models for Modeling of Interface Structures of PEC-nc-TiO_2_ Electrodes

Water (H_2_O) molecules aggregate each other via hydrogen bonding. The structures of H_2_O clusters (H_2_O)_n_ (*n =* 1~3, 6) are simulated to understand their molecular orbital energy structures (Supplementary Table S3). One of the (H_2_O)_3_ trimer is simulated to have dipole and the highest HOMO potential −6.67 eV. The HOMO distributes on the H_2_O, of which hydrogen atoms have hydrogen bond with oxygen atoms of other two H_2_O, rationalizing the most positive HOMO potential among the examined H_2_O clusters. In other words, the trimer (H_2_O)_3_ is the most oxidizable H_2_O model.

Similarly, H_2_O hydroxide ion clusters and H_2_O hydronium ion clusters are simulated (Supplementary Tables S4 and S5). In general, H_2_O hydroxide ion clusters have positive HOMO potential. With increase of H_2_O molecules, the HOMO shifts to negative potential, which means that HOMO potential is controllable by number of associating H_2_O as pH is controllable by dilution. In addition, all of H_2_O hydronium ion clusters have very negative HOMO potential −20.2~−12.36 eV with large size of LUMO configurations (Supplementary Table S5). With increase of H_2_O molecules, HOMO potential shifts to positive potential, verifying that the HOMO level is changeable as is the case of H_2_O hydroxide ion clusters, and the hydrated hydronium ion clusters become oxidizable energetically.

**Figure 3:**
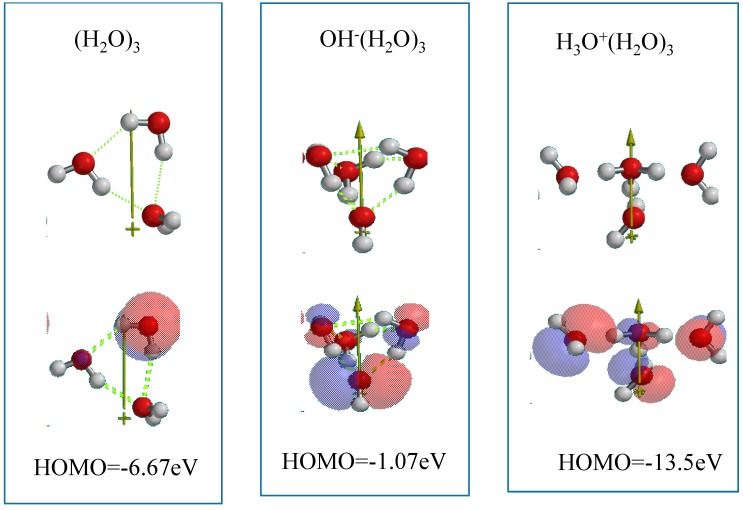
Water cluster models for DFT simulation of PEC oxidation of water molecules on PEC-nc-TiO_2_ electrodes.

With these simulations and considerations, the polar (H_2_O)_3_ and OH^−^(H_2_O)_3_ are employed for DFT-modeling of neutral interface of PEC-nc-TiO_2_ electrodes. As for hydronium ion cluster, H_3_O^+^(H_2_O)_2_, which is derived from (H_2_O)_3_, are introduced for the modeling of acidic interface of PEC-nc-TiO_2_ electrodes (Figure 3). The HOMO configurations indicate that the model clusters have electron rich parts with a wide variety of potentials ranging from −1.07 to −13.5 eV.

### 2.3. Photoelectrochemical Oxidation of H_2_O on PEC-nc-TiO_2_

**Figure 4:**
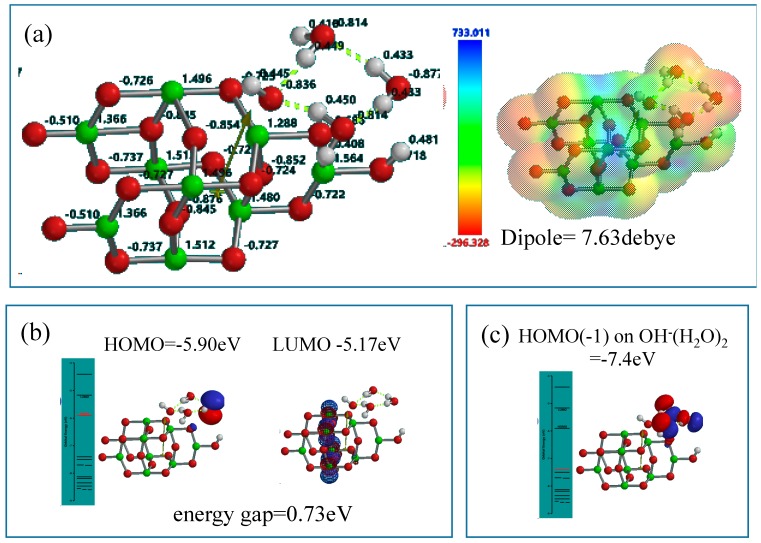
DFT-simulation of (H_2_O)_3_&OH(TiO_2_)_9_H as a stationary state of PEC-nc-TiO_2_ electrodes, (**a**) Mulliken charge and electrostatic density map; (**b**) energy structures of HOMO and LUMO; (**c**) the configuration of HOMO(−1) on (H_2_O)_3_.

As an interface model of nc-TiO_2_ electrodes, the Yamashita/Jono model, OH(TiO_2_)_9_H, is structurally frozen and three H_2_O molecules are made manually to interact via hydrogen bonding with the frozen hydroxyl group in Yamashita/Jono model. The (H_2_O)_3_-hydrogen bonded model structure is optimized by molecular mechanics (MMFF operation in Spartan), and the molecular orbital is verified by DFT-single-point simulation of an interface structure of (H_2_O)_3_&OH(TiO_2_)_9_H as a stationary model (Figure 4). Mulliken charge and electrostatic potential map indicates that negative charge locates on surface oxygen atoms of TiO_2_ and of the (H_2_O)_3_ cluster, and the more negative charge (stronger red color) on the H_2_O molecule in the cluster is worth noting in the stationary state model structure.

In the stationary model, configurations of HOMO distributes on one H_2_O of the cluster (H_2_O)_3_ unit, and LUMO inside the nc-TiO_2_ unit. Interestingly, the energy gap (0.73 eV) between HOMO and LUMO is smaller than the energy gap (2.55 eV) in the stationary state of Yamashita/Jono model. This fact suggests that nc-TiO_2_ surface binds H_2_O molecules via hydrogen bond, forming kinds of charge transfer complexes. In addition, HOMO(−1) (−7.4 eV) distributes on the whole (H_2_O)_3_ unit, implying more effective H_2_O oxidation under negative potential of −7.4 eV (Figure 4).

For comparison, only one H_2_O-associated Yamashita/Jono model, H_2_O&OH(TiO_2_)_9_H, is simulated as the simplest structure of PEC-nc-TiO_2_ electrodes (Supplementary Figure S3). The energy gap (1.75 eV) is given and the hydrogen-bonded H_2_O molecule locates HOMO(−1) at −7.8 eV. The simplest orbital energy structure verifies that nc-TiO_2_ electrodes intrinsically work as catalytic sites of H_2_O oxidation through hydrogen bonding with H_2_O molecules.

**Figure 5:**
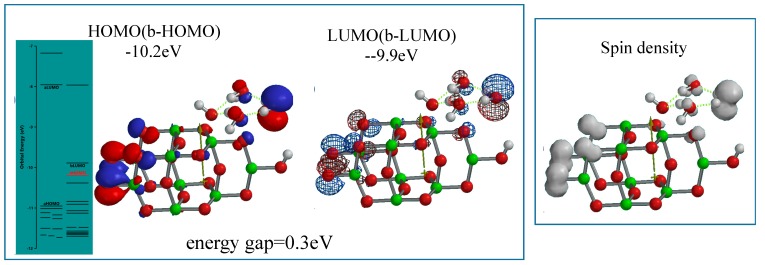
Energy structures of (H_2_O)_3_&[OH(TiO_2_)_9_H]^+^ as a radical cation model of (H_2_O)_3_-interacted PEC-nc-TiO_2_ electrode under UV-irradiated bias conditions of PEC-nc-TiO_2_ electrodes, *i.e*., a photo-energy-driven operational state model.

When nc-TiO_2_ electrodes are energy-filled by UV-irradiation at short circuit PEC conditions, photoelectron on nc-TiO_2_ is ejected to conducting grids instantly, and photocurrent become observable. The (H_2_O)_3_-associated PEC-nc-TiO_2_ model is simulated as radical cation model of [(H_2_O)_3_ &OH(TiO_2_)_9_H]^.+^ as the energy-filled structure under working conditions (Figure 5). Interestingly, the radical cation model as the working model is endothermically simulated (ΔE = 192.85 kcal/mol). The orbital energy analysis of the energy-filled model of [(H_2_O)_3_&OH(TiO_2_)_9_H]^.+^ reveals that the energy gap between HOMO and LUMO potential becomes narrow as small as 0.3 eV with largely negative HOMO (−10.2 eV) and LUMO (−9.9 eV) potential. The HOMO and LUMO distribute in the model with almost the same configurations, and that the spin (unpaired electron) density distributes with the same configuration as the HOMO and LUMO.

The orbital energy analysis of the energy-filled Yamashita/Jono model of [OH(TiO_2_)_9_H]^.+^ reveals that the energy gap is pretty narrow (0.7 eV) (Supplementary Table S2). These facts suggest that self-organization of Yamashita/Jono model will give photoconductive nc-TiO_2_ electrodes when the PEC cell is kept at negative oxidation potential at short circuit conditions and energized by band gap excitation. In fact, the sharp rise in photoconductivity of nc-TiO_2_ electrodes was reported and discussed as an insulator-metal (Mott) transition in a donor band of anatase TiO_2_ [11]. It is also worth noting that in studies on dye-sensitized nc-TiO_2_ solar cells (DSC), adsorption of cationic species like tetrabutylammonium cation and sensitizing dye molecules enhanced electron transport in nc-TiO_2_ electrodes [12,13]. Accordingly, the photo-enhanced electron transport is now verified as a key function of nc-TiO_2_ electrodes not only in DSC but also in PEC H_2_O oxidation.

The HOMO configuration on the H_2_O unit at the stationary state of the model, and the spin density distribution on the (H_2_O)_3_ unit at the energy-filled working state strongly suggest that PEC-nc-TiO_2_ electrodes provides catalytic binding sites of H_2_O. The same functions of PEC nc-TiO_2_ electrodes are confirmed by the molecular orbital simulation of the energy-filled [H_2_O&OH(TiO_2_)_9_H]^+^ model (Tables S1 and S2, Figure S4). The narrowed energy gap (0.3 eV), the comparable configurations of HOMO, LUMO and spin density are quite comparable with those of [(H_2_O)_3_&OH(TiO_2_)_9_H]^+^.

### 2.4. DFT Simulation of H_2_O Oxidation to Hydrogen Peroxide

In PEC H_2_O oxidation on nc-TiO_2_ electrodes, bias potential is essential to start H_2_O oxidation. The HOMO potential of Yamashita/Jono model, −7.18 eV and the average HOMO potential of H_2_O clusters, −7.48 eV (Supplementary Table S3) verify that the bias potential >0.3V is at lease required for PEC H_2_O oxidation under neutral working conditions. On the other hand, the HOMO potential (−10.2 eV) of the working model of [(H_2_O)_3_&OH(TiO_2_)_9_H]^+^ predicts that successive oxidation should occur under more negative bias potential (>2.72 eV) for H_2_O oxidation to oxygen through formation of hydrogen peroxide.

**Figure 6:**
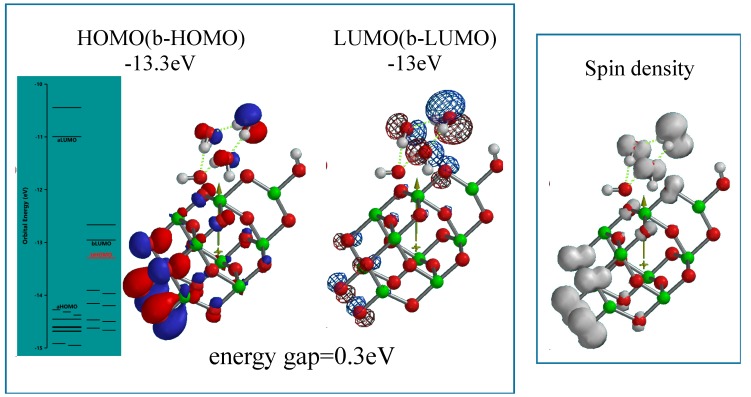
Energy structures of [(H_2_O)_3_]&OH(TiO_2_)_9_H]^..++^ as the model of two electron oxidation state of PEC-nc-TiO_2_ electrodes, *i.e*., the diradical-dication state of the electrodes.

In order to verify whether hydroxyl radical may form successively on PEC-nc-TiO_2_ electrodes, the two-electron oxidation structure of [H_2_O&OH(TiO_2_)_9_H]^..++^ is simulated as dication-diradical model as another working interface model of PEC-nc-TiO_2_ electrodes. The more energy-filled model is endothermically (ΔE = 458.43 kcal/mol) simulated as powerful working model (Figure 6). The energy gap (0.3 eV) and the configuration of HOMO and LUMO are confirmed to verify that such largely energized PEC-nc-TiO_2_ electrodes keep photoconductivity with keeping high oxidation potential. The spin density distributes on the (H_2_O)_3_ unit, suggesting that two-electron H_2_O oxidation occurs successively on the catalytic site on nc-TiO_2_ electrodes.

**Scheme 1:**
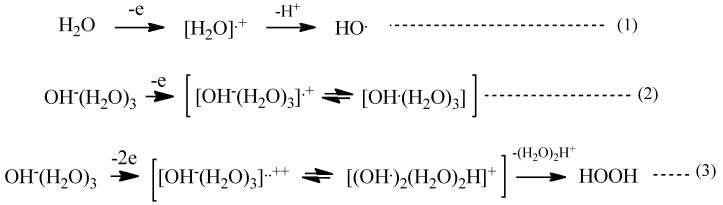
PEC-H_2_O oxidation to hydrogen peroxide as a precursor of oxygen molecule.

With these simulation analyses, step-wise PEC-H_2_O oxidation is shown in Scheme 1. One-electron oxidation of H_2_O molecule yields radical cation of H_2_O (H_2_O^+^) and the removable of proton from the radical cation (deprotonation) leads to hydroxyl radical (HO) (Equations (1) and (2) in Scheme 1). When H_2_O hydroxide ion cluster of OH^−^(H_2_O)_3_ undergoes further oxidation, another hydroxyl radical favorably forms in neighbor on PEC-nc-TiO_2_ electrodes, and efficient and effective formation of hydrogen peroxide occurs (Equation (3) in Scheme 1).

**Figure 7:**
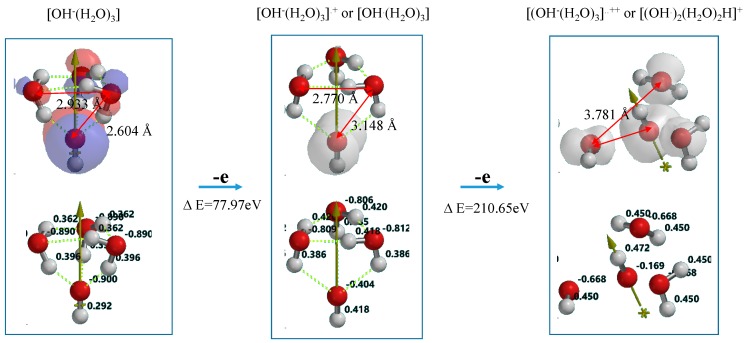
DFT-simulation of step-wise oxidation of the hydroxide ion cluster model of [OH^−^(H_2_O)_3_].

Figure 7 shows DFT-simulation results of oxidation of OH^−^(H_2_O)_3_ to [OH^−^(H_2_O)_3_]^.+^ or [OH^.^(H_2_O)_3_] as one-electron oxidation products (Equation (2)), and to [OH^−^(H_2_O)_3_]^..++^ or [(OH^.^)_2_(H_2_O)_2_H]^+^ as two-electron oxidation products. They are simulated endothermically, suggesting that they are in energy filled states (Supplementary Table S6). In the equilibrium geometry of [OH^.^(H_2_O)_3_]^.+^, the spin density distributes only on the hydroxyl group, and the Mulliken charge on hydroxyl group decreases largely from −0.900 to −0.404. Thus the one-electron oxidation product has the structure of [OH^.^(H_2_O)_3_] rather than [OH^−^(H_2_O)_3_]^.+^.

On the other hand, the two-electron oxidation product shows that the spin density distributes on hydroxyl group and the (H_2_O)_3_ units. The Mulliken charge on them decreases from −0.806 to −0.668. The distance between hydroxyl group and H_2_O is shortened from 3.148 Å to 2.239 Å and the hydrogen bonds observed in [OH^−^(H_2_O)_3_]^.+^ disappear. In addition, the hydrogen-oxygen bond distance of H_2_O (0.976 Å) in [OH^−^(H_2_O)_3_]^..++^ is quite comparable with that (0.988 Å) in [OH^−^(H_2_O)_3_]^.+^. The H_2_O components in the most energy-filled cluster [OH^−^(H_2_O)_3_] ^++^ should be tightly aggregated one another. Detachment of (H_2_O)_3_H^.+^ from [(OH^.^)_2_(H_2_O)_2_H^+^] leaves two hydroxyl radical in neighbor, yielding hydrogen peroxide as a precursor of O_2_ molecule (Equation (3) in Scheme 1). The oxidation of neutral H_2_O is verified to occur initially in photocatalytic processes to give effectively hydrogen peroxide on PEC nc-TiO_2_ electrodes.

### 2.5. Verification of the Sakka’s PEC H_2_O Oxidation under Acidic Conditions

The stationary model of H_3_O^+^(H_2_O)-associated structure of H_3_O^+^(H_2_O)&OH(TiO_2_)_9_H and two kinds of energy filled models, [H_3_O^+^(H_2_O)&OH(TiO_2_)_9_H]^.+^ and [H_3_O^+^(H_2_O)&OH(TiO_2_)_9_H]^.++^ are simulated as an interface model of PEC-nc-TiO_2_ electrodes under Sakka’s acidic conditions (Supplementary Figures S5 and S6). However, the energy gap are rather wider and the spin density does not localize on H_3_O^+^(H_2_O) in the most energized state of [H_3_O^+^(H_2_O)&OH(TiO_2_)_9_H]^.++^.

**Figure 8:**
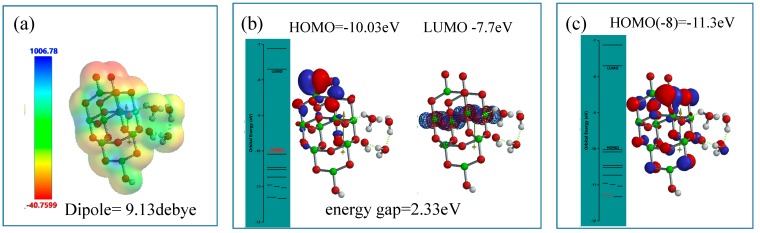
DFT-simulation of H_3_O^+^(H_2_O)_2_&OH(TiO_2_)_9_H as a model of hydronium ion clusters on PEC-nc-TiO_2_ electrodes, (**a**) electrostatic potential map; (**b**) structures of HOMO and LUMO; (**c**) Configuration of HOMO(−8).

The hydronium ion cluster, H_3_O^+^(H_2_O)_2_ represent less acidic than H_3_O^+^(H_2_O) (Supplementary Table S5). The stationary states of H_3_O^+^(H_2_O)_2_-associated structure of H_3_O^+^(H_2_O)_2_&OH(TiO_2_)_9_H is simulated as an interface model under Sakka’s less acidic conditions, and analyzed as well in view of molecular orbital energy structure (Figure 8).

Differently from the modeling for the neutral PEC H_2_O oxidation, electrostatic potential map indicates that negative charge locates much more on the nc-TiO_2_ unit rather than the H_2_O unit, and HOMO distributes only on oxygen atoms in the nc-TiO_2_ unit. The energy gap 2.33 eV implies weak association of acidic H_2_O on nc-TiO_2_ electrodes. The orbital energy indicates that HOMO(−8) distributes slightly on the H_3_O^+^(H_2_O)_2_ unit with very negative potential of −11.3 eV.

**Figure 9:**
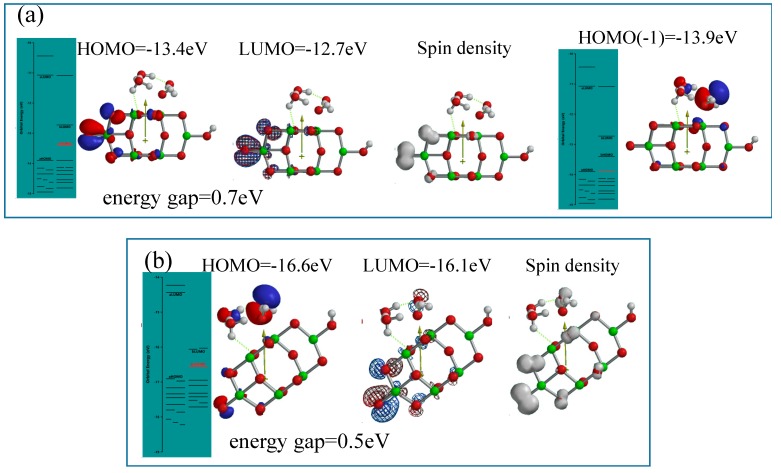
DFT-simulation of oxidation states of H_3_O^+^(H_2_O)_2_&OH(TiO_2_)_9_H, (**a**) energy structures of the one-electron oxidation state, [H_3_O^+^(H_2_O)_2_&OH(TiO_2_)_9_H]^.+^; (**b**) energy structures of the two-electron oxidation state, [H_3_O^+^(H_2_O)_2_&OH(TiO_2_)_9_H]^..++^.

The acidic interface model of [H_3_O^+^(H_2_O)_2_&OH(TiO_2_)_9_H] is simulated to [H_3_O^+^(H_2_O)_2_& OH(TiO2)_9_H]^.+^ as the radical cation of the one-electron oxidation state, and to [H_3_O^+^(H_2_O)_2_& OH(TiO2)_9_H]^..++^ as the diradical-dication model of the two electron oxidation sate (Figure 9). The former radical cation model reveals that configurations of HOMO, LUMO and spin density distribute on the nc-TiO_2_ unit and not on the H_3_O^+^(H_2_O)_2_, and the energy gap 0.7eV is not favorable in view of photoconductivity compared to that under neutral conditions. However, HOMO(−1) distributes on the (H_2_O)_2_ unit with orbital potential of −13.9 eV.

As for the latter dication-diradical model, the energy gap 0.5 eV and HOMO potential, −16.6 eV are given, and the spin density distributes on H_3_O^+^(H_2_O)_2_ unit. Accordingly, the DFT-based orbital energy structure verifies that PEC H_2_O oxidation occurs even under acidic conditions, when nc-TiO_2_ electrodes are energized by bias potential under energy-gap UV irradiation.

## 3. Experimental Section

DFT calculations were performed using the B3LYP exchange-correlation functional and the 6-31G(d) basis set with *Spartan’14* (Wavefunction, Inc. Irvine, CA, USA) installed on VAIO Model SVP132A1CN, Intel(R) core(TM)i7-4500U CPU and on VAIO PC-Z (Intel core 2 Duo processor T9900, system memory (RAM) 8G and hard disk drive, SSD 128, 2GB).

Molecular mechanic optimization (e.g., Merck Molecular Force Factor (MMFF) operation in Spartan program) and DFT (B3LYP 6-31G*) modeling determine molecular orbital structure of equilibrium geometry as an inter-atomic potential model [7,8]. In the case of Spartan program, molecular orbital energy structures (HOMO(0~9), LUMO(0~1), their configurations, electrostatic potential map, spin (unpaired electron of radical) density are visualized by graphic conveniently.

As for interface energy-filled model structures with unpaired electron, *i.e.*, radical cations, orbital energy diagrams are shown in two ways, affixed ‘a-’ and ‘b-’ because the radical cations have two available wave functions. The ‘b’-HOMO and ‘b’-LUMO are employed, since ‘β’-HOMO configurations are almost the same as spin density configurations. Electron energy gap of the radical cation components is obtained from energy difference between ‘b’-HOMO and ‘b’-LUMO. Mulliken charge, spin densities and their maps are informative for theoretical understandings of energy structures of energy-filled molecular orbitals of nc-TiO_2_ interfaces.

The anatase nc-TiO_2_ model structure has a pretty large size of OH(Ti_9_O_18_)H and is named as Yamashita/Jono model. The nc-TiO_2_ model structure is refined as described in Figure S7. As for orbital configurations, HOMO is shown by solid or solid transparent, LUMO by mesh, and spin density by white solid or white solid transparent. As for color in electrostatic potential map, red is negative, green neutral and blue positive qualitatively. Formation energy (ΔE) of key model molecules is determined from total energy (E) of their related components in Supplementary Tables S1–S3.

## 4. Conclusions

DFT-based modeling enables to verify molecular orbital level interfacial structures of photo-electrochemically energized nc-TiO_2_ electrodes. Although nc-TiO_2_ electrodes are composed of nc-TiO_2_ particles with average size 25 nm, Yamashita/Jono nc-TiO_2_ model (length size = ~1 nm) is large enough to model nc-TiO_2_ electrodes because the model may self-aggregate to larger sizes through hydrogen bond and van der Waals and Coulombic interactions. Water (H_2_O) cluster models (H_2_O)_3_ and H_3_O^+^(H_2_O)_2_ are appropriate to bind Yamashita/Jono model cluster, providing interfacial PEC-nc-TiO_2_ electrode structures at neutral and acidic H_2_O conditions.

Molecular orbital analyses of the stationary and the working PEC-nc-TiO_2_ cluster models reveal that H_2_O clusters are adsorbed effectively (catalytically) via hydrogen bonding to PEC-nc-TiO_2_ electrodes at stationary state, and that the conductivity of PEC-nc-TiO_2_ electrodes is enhanced without loosing oxidation potential, leading to successive water oxidation to oxygen molecules through hydrogen peroxide in PEC cells. The molecular modeling of nc-TiO_2_ electrodes in PEC cells verifies that the photo-induced conductivity is the most important driving force of PEC H_2_O oxidation on nc-TiO_2_ electrodes. The DFT-verified photoconductivity is true for understanding of photocatalysis of Pt-deposited nc-TiO_2_ particles and dye-sensitized nc-TiO_2_ solar cells, rationalizing their remarkable efficiencies and effectiveness.

## Supplementary Materials

Supplementary materials can be accessed at: http://www.mdpi.com/1420-3049/20/06/9732/s1.

## References

[1] Grätzel M. (2001). Photoelectrochemical cells. Nature.

[2] Hashimoto K., Irie H., Fujishima A. (2006). Photoelectrochemical cells. JSAP Int..

[3] Yoko T., Kamiya K., Sakka S. (1987). Photoelectrochemical Properties of TiO_2_ Films Prepared by the Sol-Gel Method. Yogyo Kyoukai-Shi.

[4] Kanemoto M., Hosokawa H., Wada Y., Murakoshi K., Yanagida S., Sakata T., Mori H., Ishikawa M., Kobayashi H. (1996). Semiconductor photocatalysis. Part 20. Role of surface in the photoreduction of carbon dioxide catalysed by colloidal ZnS nanocrystallites in organic solvent. J. Chem. Soc. Faraday Trans..

[5] Manseki K., Yu Y., Yanagida S. (2013). A phenyl-capped aniline tetramer forZ907/*tert*-butylpyridine-based dye-sensitized solar cells and molecular modelling of the device. Chem. Commun..

[6] Yanagisawa S., Yasuda T., Inagaki K., Morikawa Y., Manseki K., Yanagida S. (2013). Intermolecular Interaction as the Origin of Red Shifts in Absorption Spectra of Zinc-Phthalocyanine from First-Principles. J. Phys. Chem. A.

[7] Hehre W.J. (2003). Chapter 19. Application of Graphical models in book. A Guide to Molecular Mechanics and Quantum Chemical Calculations.

[8] Hoffmann R. (1988). The frontier orbital perspective in book SOLID and SURFACES. A Chemist’s View of Bonding in Extended Structures.

[9] Agrawal S., English N.J., Thampi K.R., MacElroy J.M.D. (2012). Perspectives on *ab initio* molecular simulation of excited-state properties of organic dye molecules in dye-sensitised solar cells. Phys. Chem. Chem. Phys..

[10] Jono R., Fujisawa J., Segawa H., Yamashita K. (2011). Theoretical Study of the Surface Complex between TiO_2_ and TCNQ Showing Interfacial Charge-Transfer Transitions. J. Phys. Chem. Lett..

[11] Wahl A., Augustynski J. (1998). Charge Carrier Transport in Nanostructured Anatase TiO_2_ Films Assisted by the Self-Doping of Nanoparticles. J. Phys. Chem. B.

[12] Kambe S., Nakade S., Kitamura T., Wada Y., Yanagida S. (2002). Influence of the Electrolytes on Electron Transport in Mesoporous TiO_2_-Electrolyte Systems. J. Phys. Chem. B.

[13] Nakade S., Saito Y., Kubo W., Kanzaki T., Kitamura T., Wada Y., Yanagida S. (2003). Enhancement of electron transport in nano-porous TiO_2_ electrodes by dye adsorption. Electrochem. Commun..

