# Supplementary material for: Theoretical Verification of Photoelectrochemical Water Oxidation Using Nanocrystalline TiO2 Electrodes
Source: Molecules. 2015 May 27;20(6):9732–44. doi: 10.3390/molecules20069732 (PMC6272316; doi:10.3390/molecules20069732)
Supplement: Supplementary file 1 [file molecules-20-09732-s001.pdf]

Supplementary Materials

Table S1. DFT simulation for H_2O-cluster association on PEC-nc-TiO_2 electrodes using Yamashita/Jono model.

Stationary models of PEM-nc-TiO_2 electrodes	E LUMO (eV)	E HOMO (eV)	energy gap (eV)	Dipole (debye)	E (kcal/mol)	DE* (kcal/mol)
$(TiO_2)_9$	-5.23	-6.27	1.04	2.39	-5647685.57	-
$(TiO_2)_9H$	-7.99	-10.24	2.25	10.98	-5647943.44	-257.87
$OH(TiO_2)_9H$	-4.63	-7.18	2.55	10.44	-5695690.26	-231.28*
$H_2O\&OH(TiO_2)_9H$	-4.86	-6.61	1.75	7.46	-5743633.92	3.72*
$(H_2O)_3\&\ OH(TiO_2)_9H$	-5.17	-5.90	0.73	7.63	-5839550.06	-17.66*
$H_3O^+(H_2O)\ \&OH(TiO_2)_9H$	-7.69	-10.02	2.33	9.97	-5791820.62	-59.81*
$H_3O^+(H_2O)_2\&OH(TiO_2)_9H$	-7.7	-10.03	2.33	9.13	-5839787	-78.81

*determined from the total energy of $(TiO_2)_9H^+$ and water-derived species.

Table S2. DFT simulation for H_2O oxidation on PEC-nc-TiO_2 electrodes using Yamashita/Jono model.

Working models of PEM-nc-TiO_2 electrodes	E LUMO (b- LUMO) (eV)	E HOMO (b-HOMO) (eV)	energy gap (eV)	E a- LUMO (eV)	E a-HOMO (eV)	Dipole (debye)	E (kcal/mol)	DE* (kcal/mol)
$[OH(TiO_2)_9]^{\cdot+}$	-9.8	-10.5	0.7	-8.01	-10.96	4.04	-5695494.29	195.97
$H_2O\&OH(TiO_2)_9H]^{\cdot+}$	-9.9	-10.2	0.3	-7.95	-11.05	3.38	-5743437.59	195.94
$[(H_2O)_3\&OH^-(TiO_2)_9H]^{\cdot+}$	-9.9	-10.2	0.3	-7.96	-10.95	5.23	-5839357.21	192.85
$[(H_2O)_3\&OH^-(TiO_2)_9H]^{\cdot\cdot++}$	-13	-13.3	0.3	-10.99	-14.28	6.67	-5839091.63	458.43
$[H_3O^+(H_2O)\&OH(TiO_2)_9H]^{\cdot+}$	-12.7	-13.4	0.7	-11.08	-14.14	3.25	-5791558.58	262.04
$[H_3O^+(H_2O)\ \&OH(TiO_2)_9H]^{\cdot\cdot++}$	-16.1	-17	0.9	-14.5	-16.97	9.33	-5791218.26	602.36
$[H_3O^+(H_2O)_2\&OH(TiO_2)_9H]^{\cdot+}$	-12.7	-13.4	0.7	-11.08	-13.9	6.58	-5839524.71	262.29
$[H_3O^+(H_2O)_2\&OH(TiO_2)_9H]^{\cdot\cdot++}$	-16.1	-16.6	0.5	-14.43	-16.91	12.86	-5839184.68	602.32

Table S3. DFT-simulation of H_2O clusters for molecular orbitals and their energy structures

Water clusters	E LUMO (eV)	E HOMO (eV)	Energy gap (eV)	Dipole (debye)	E (kcal/mol)	DE (kcal/mol)
H_2O	1.7	-7.92	9.62	2.1	-47947.38	-
$(H_2O)_2$	1.1	-7.21	8.31	1.72	-95902.45	-7.69
$(H_2O)_3$	0.5	-6.67	7.17	5.36	-143856.58	-14.44
sym $(H_2O)_3$	1.66	-7.67	9.33	0	-143861.99	-19.85
$(H_2O)_6$	1.51	-7.95	9.46	0	-287750.01	-65.73

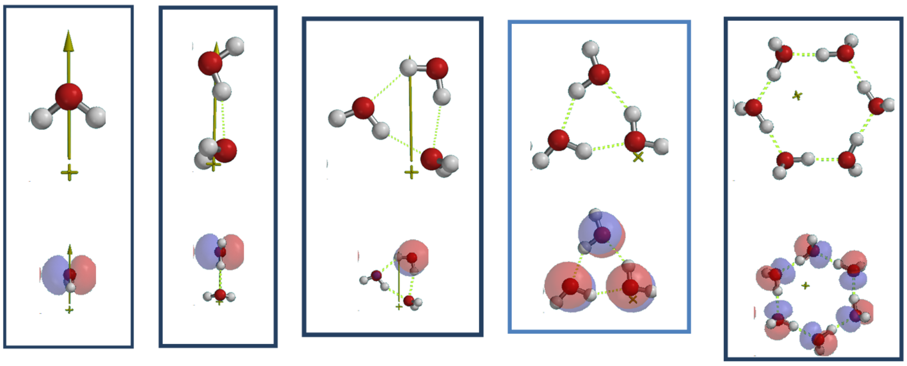

Table S4. DFT-simulation of H_2O hydroxide ion clusters for molecular orbitals and their energy structures.

Hydroxide ion clusters	E LUMO (eV)	E HOMO (eV)	Energy gap (eV)	Dipole (debye)	E (kcal/mol)	Δ E (kcal/mol)
OH^-	11.25	4.38	6.87	1.19	-47515.54	-
OH^-H_2O	8.75	1.06	8.75	0.78	-95508.53	-45.61
$OH^-(H_2O)_2$	7.56	-0.14	7.56	1.87	-143490.2	-79.9
$OH^-(H_2O)_3$	7.29	-1.07	7.29	0.94	-191465.3	-107.62
$OH^-(H_2O)_6$	5.49	-2.85	8.34	1.71	-335374.44	-174.62

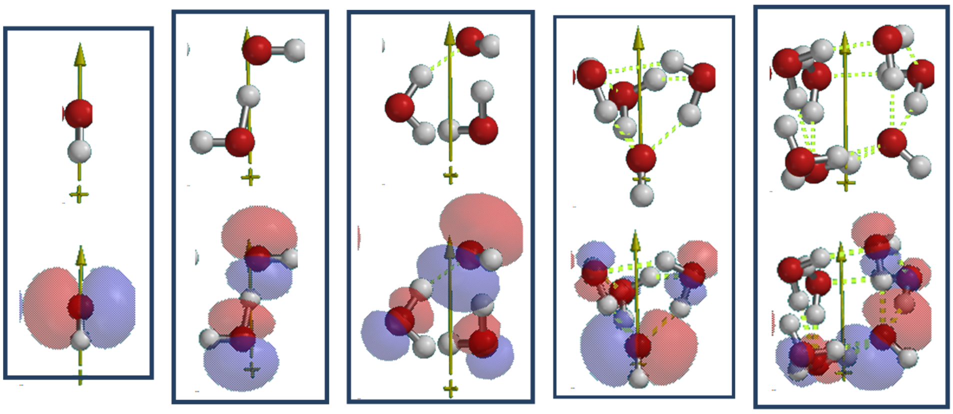

Table S5. DFT-simulation of H_2O hydronium ion clusters for molecular orbitals and their energy structures.

Hydronium ion clusters	E LUMO (eV)	E HOMO (eV)	Energy gap (eV)	Dipole (debye)	E (kcal/mol)	DE (kcal/mol
H_3O^+	-7.31	-20.2	12.89	1.7	-48123.17	-
$H_3O^+H_2O$	-5.08	-15.9	10.82	1.4	-96110.71	-40.16
$H_3O^+(H_2O)_2$	-3.98	-14.29	10.31	1.78	-144086.31	-68.38
$H_3O^+(H_2O)_3$	-3.26	-13.5	10.24	0.52	-192057.69	-92.38
$H_3O^+(H_2O)_6$	-3.23	-12.36	9.13	5.25	-335957.21	-149.76

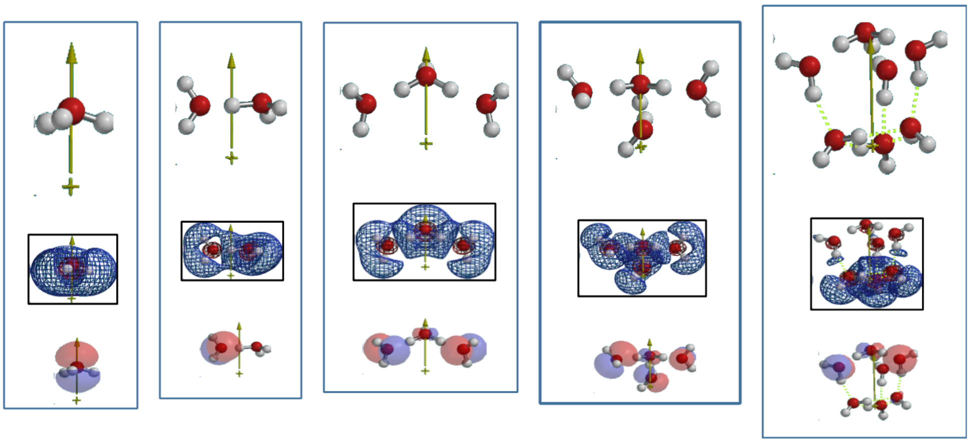

Table S6. DFT-simulation for step-wise oxidation of H_2O hydronium ion cluster.

hydroxide ion cluster	E LUMO (b-LUMO) (eV)	E HOMO (b-HOMO) (eV)	Energy gap (eV)	E a- LUMO (eV)	E a-HOMO (eV)	Dipole (debye)	E (kcal/mol)	Δ E (kcal/mol)
$[OH^{\cdot}(H_2O)_3]^{+}$	-5.6	-7.8	2.2	0.54	-7.78	6.37	-191387.33	77.97
$[(OH^{\cdot})_2(H_3O)^{+}(H_3O)_2]^{\cdot++}$	-9.6	-13.1	3.5	-3.82	-13.81	2.02	-191176.68	210.65

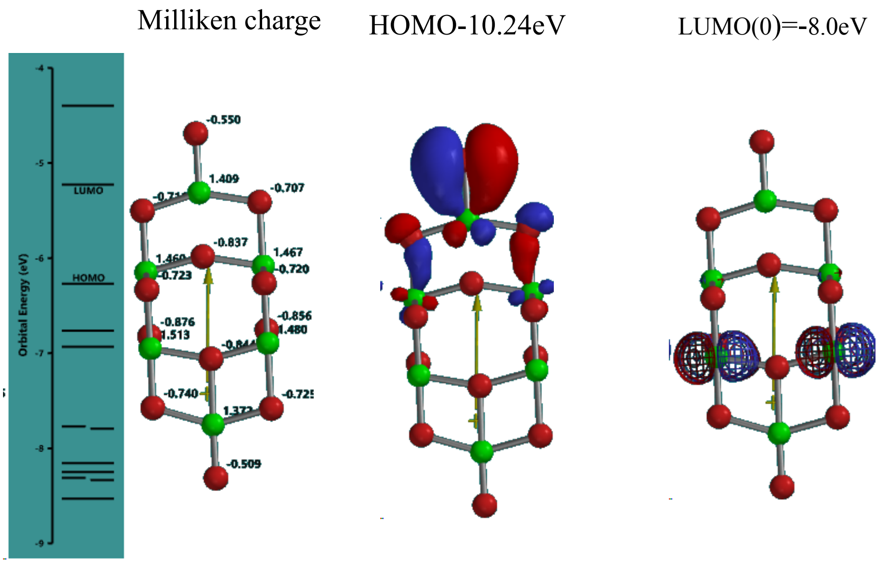

Figure S1. DFT-simulated molecular orbitals and energy structures of a surface fragment $(TiO_2)_9$ of Yamashita/Jono model.

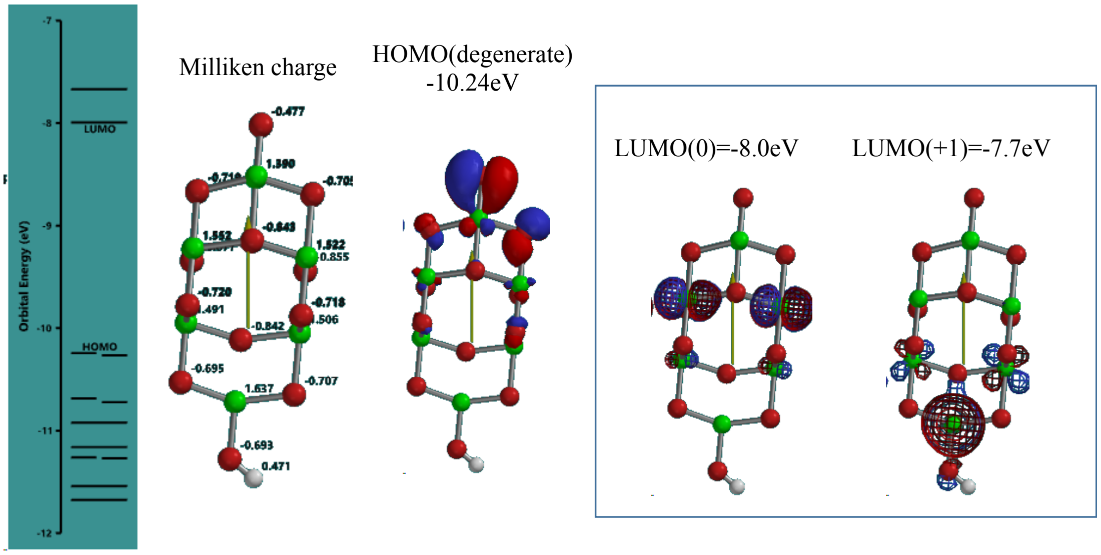

Figure S2. DFT-simulated molecular orbitals and energy structures of a fragment $(TiO_2)_9H$ from of Yamashita/Jono model.

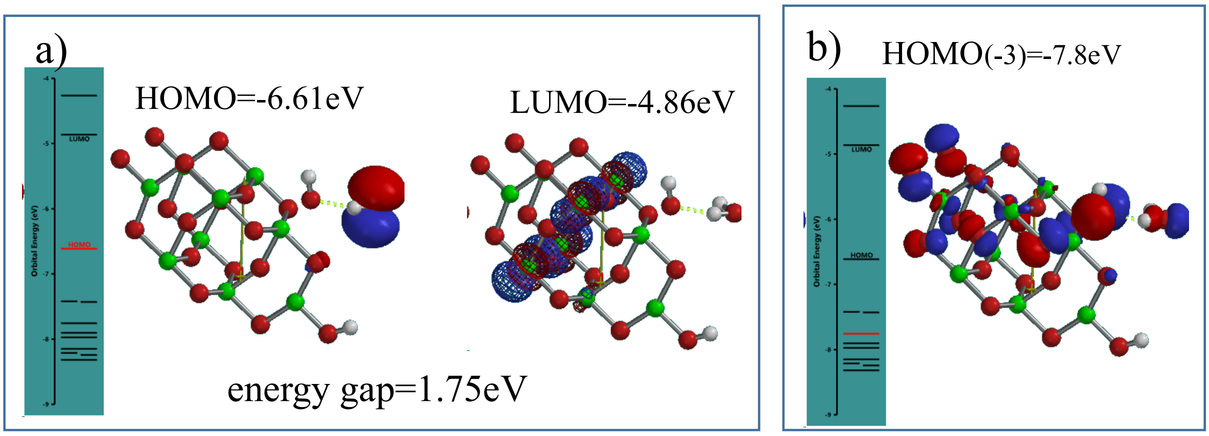

Figure S3. DFT-simulation of H_2O-adsorbed PEC-nc-TiO_2 electrodes, H_2O&$OH(TiO_2)_9H$. (**a**) Energy structures of HOMO and LUMO, (**b**) the configuration of HOMO(−3) on H_2O.

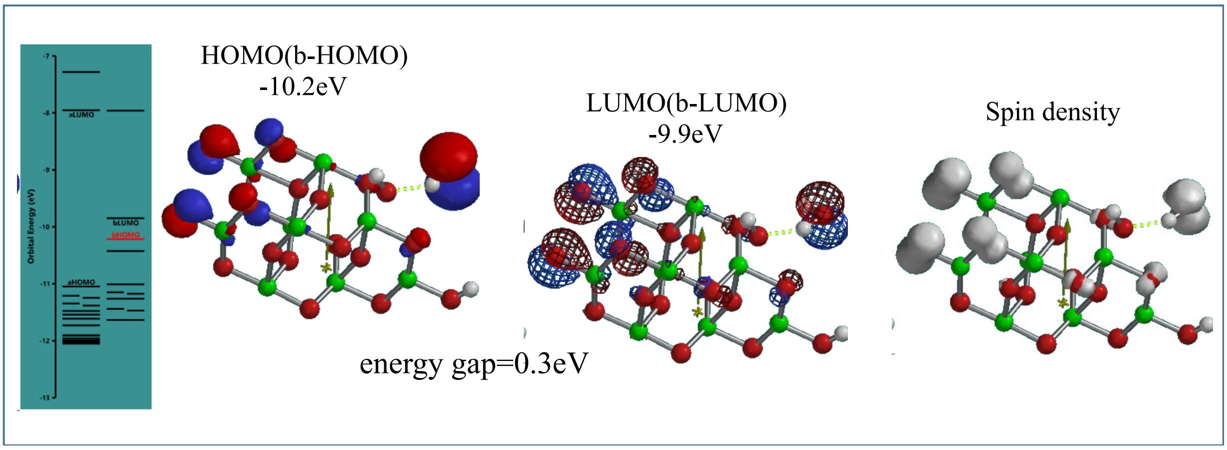

Figure S4. DFT-simulation of $[H_2O\&OH(TiO_2)_9H]^{+}$ as a cation radical model of H_2O-interacted PEC-nc-TiO_2 electrode under UV-irradiated bias conditions.

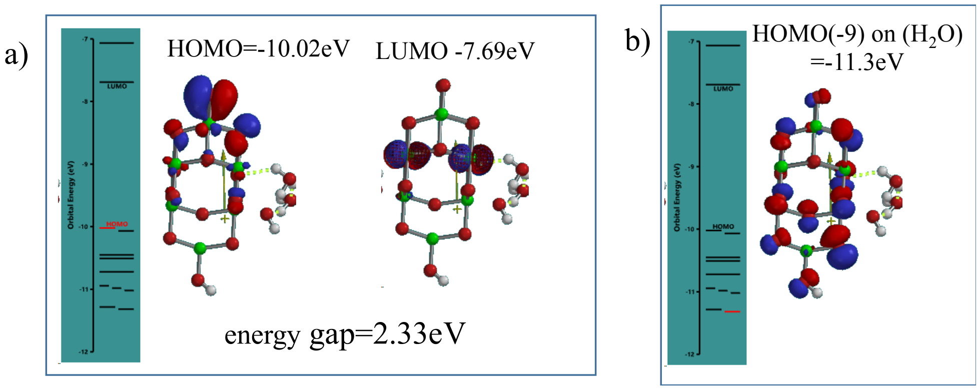

Figure S5. DFT-simulation of $H_3O^{+}(H_2O)\&OH(TiO_2)_9H$ as a model of $H_3O^{+}(H_2O)$-adsorbed PEC-nc-TiO_2 electrodes, (**a**) Energy structures of HOMO and LUMO, (**b**) the configuration of HOMO(−7).

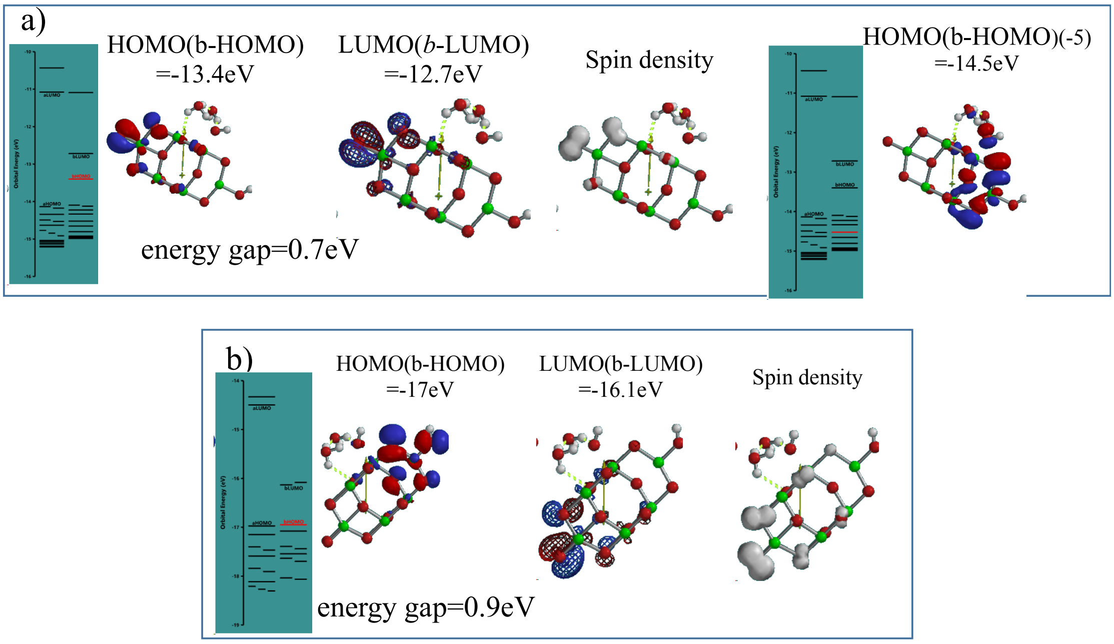

Figure S6. Energy structures of one- and two-electron oxidation states of $H_3O^+(H_2O)$&$OH(TiO_2)_9H$, (**a**) the one-electron oxidation state: $[H_3O^+(H_2O)\&OH(TiO_2)_9H]^{\cdot+}$, (**b**) the two-electron oxidation state: $[H_3O^+ (H_2O)\&OH(TiO_2)_9H]^{\cdot\cdot++}$.

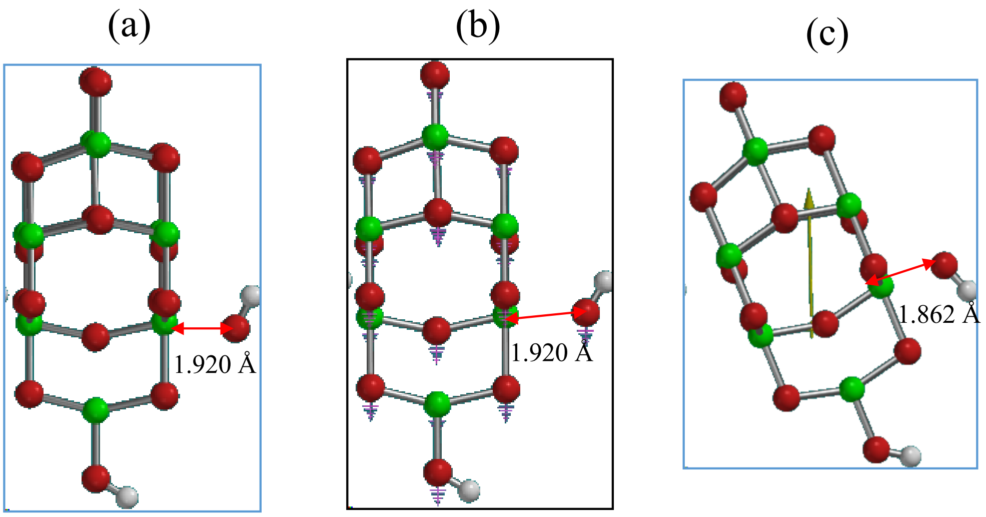

(a) Yamashita/Jono model, (b) The bonding between O and Ti atom is all frozen and optimized by MMFF. (c) the bond between a hydroxyl group and Ti atom is broken, and the heavy atom is all frozen and optimized by MMFF and the structure is simulated for the equilibrium geometry after the hydroxyl group is thawed and the optimized Yamashita/Jono model is obtained. Hereafter, the heavy atom in the model is all frozen and introduced to DFT simulation of PEC-nc-TiO_2 electrodes

Figure S7. Optimization of Yamashita/Jono model, $OH(TiO_2)_9H$.
